# Inertial Measures of Motion for Clinical Biomechanics: Comparative Assessment of Accuracy under Controlled Conditions – Changes in Accuracy over Time

**DOI:** 10.1371/journal.pone.0118361

**Published:** 2015-03-26

**Authors:** Karina Lebel, Patrick Boissy, Mathieu Hamel, Christian Duval

**Affiliations:** 1 Faculty of Medicine and Health Sciences, Orthopedic Service, Department of Surgery, Université de Sherbrooke, Sherbrooke, Quebec, Canada; 2 Research Center on Aging, Sherbrooke, Quebec, Canada; 3 Interdisciplinary Institute for Technological Innovation (3IT), Université de Sherbrooke, Sherbrooke, Quebec, Canada; 4 Department of Kinesiology, Université du Québec à Montréal, Montreal, Quebec, Canada; 5 Centre de Recherche Institut Universitaire de Gériatrie de Montréal, Montreal, Quebec, Canada; Ludwig-Maximilian University, GERMANY

## Abstract

**Background:**

Interest in 3D inertial motion tracking devices (AHRS) has been growing rapidly among the biomechanical community. Although the convenience of such tracking devices seems to open a whole new world of possibilities for evaluation in clinical biomechanics, its limitations haven’t been extensively documented. The objectives of this study are: 1) to assess the change in absolute and relative accuracy of multiple units of 3 commercially available AHRS over time; and 2) to identify different sources of errors affecting AHRS accuracy and to document how they may affect the measurements over time.

**Methods:**

This study used an instrumented Gimbal table on which AHRS modules were carefully attached and put through a series of velocity-controlled sustained motions including 2 minutes motion trials (2MT) and 12 minutes multiple dynamic phases motion trials (12MDP). Absolute accuracy was assessed by comparison of the AHRS orientation measurements to those of an optical gold standard. Relative accuracy was evaluated using the variation in relative orientation between modules during the trials.

**Findings:**

Both absolute and relative accuracy decreased over time during 2MT. 12MDP trials showed a significant decrease in accuracy over multiple phases, but accuracy could be enhanced significantly by resetting the reference point and/or compensating for initial Inertial frame estimation reference for each phase.

**Interpretation:**

The variation in AHRS accuracy observed between the different systems and with time can be attributed in part to the dynamic estimation error, but also and foremost, to the ability of AHRS units to locate the same Inertial frame.

**Conclusions:**

Mean accuracies obtained under the Gimbal table sustained conditions of motion suggest that AHRS are promising tools for clinical mobility assessment under constrained conditions of use. However, improvement in magnetic compensation and alignment between AHRS modules are desirable in order for AHRS to reach their full potential in capturing clinical outcomes.

## Introduction

Functional mobility is an important aspect of a person’s quality of life. Its evaluation therefore plays an important role in numerous clinical decisions throughout the continuum of care, in various fields of practice ranging from rehabilitation to geriatrics. Because of their simplicity, self-report questionnaires, scales and performance-based clinical tests (e.g. Timed-Up and Go) are most commonly used for mobility evaluation. Where the resources are available, evaluations may also be based on instrumented performance tests such as 3D capture of joint motion using traditional optical or magnetic tracking systems. Recently, interest in 3D inertial motion tracking devices, also referred to as Attitude and Heading Reference Systems (AHRS), has been growing rapidly among the biomechanical experts community. AHRS are composed of a set of inertial sensors (accelerometers, gyroscopes and magnetometers) whose outputs are fed into a fusion algorithm in order to determine the orientation of a rigid body in a global reference frame, defined from gravity and magnetic North. AHRS offer a flexible and lower cost alternative to traditional motion capture systems and their convenience opens up possibilities for different applications in clinical biomechanics. However, this technology also has limitations, which have not been extensively documented.

Several recent studies explored the validity of AHRS orientation measurement on market-available systems. A first category of studies focused on the assessment of the technological validity under semi-controlled conditions. Picerno et al. evaluated the ability of multiple modules to determine a common orientation using a Plexiglas plank on which 9 units of the same AHRS model were aligned [1]. The plank was then rotated 90° at a time, and the orientation measured by all units were recorded for each orientation. Under such conditions, the authors observed a worst-case discrepancy of 5.7° between the measured orientations, which led them to conclude that the modules tested defined their orientation differently. Cutti et al. also made use of a rigid plate on which they affixed four modules to assess orientation errors between pairs of modules during static and dynamic trials [2]. Using this setup, the authors were able to identify an effect of velocity and direction of rotation on the precision of the orientation measurement, revealing a worst-case orientation error of 5.4° and 11.6° for mean rotation velocities of 180 °/s and 360°/s respectively. All studies described above considered relatively short acquisitions (< 15s), making it very hard to evaluate the stability of the accuracy of orientation measurement in time. Another category of studies tried to contextualize the validity assessment in a specific biomechanical setting. For example, Brennan et al. used an instrumented Gimbal modelling a right knee to assess accuracy of a pair of AHRS [3]. Comparison of the AHRS measured orientation to a potentiometer gold standard revealed a root-mean-square error of 3.2° in flexion/extension, 3.4 ° for abduction/adduction and 2.9 ° for internal/external rotation. All of the above-mentioned studies revealed important information regarding limits of the systems tested. However, those studies were performed on a single system at a time and based on manually-controlled, unreproducible conditions. Conditions and variables also varied so much between the different studies that it is difficult to compare the conclusions.

More recently, Lebel et al. employed a velocity-controlled Gimbal table to assess accuracy of three different AHRS systems under various controlled conditions [4]. The AHRS were tested one at a time on the Gimbal table using multiple units of the same system. A series of static trials followed by single- and multi-axes trials were performed at different speeds to assess for the systems’ absolute (i.e. 1 module vs optical gold standard) and relative (i.e. pair of modules) accuracy. Using such setup, the authors observed that all systems revealed a mean absolute error varying between 0.5° and 3.1° and a mean relative accuracy between 2° and 7° under slow motions. Accuracy was significantly affected by velocity during sustained motion for all AHRS although the extent of that effect varied across the systems.

All of the aforementioned studies were conducted over a specific, short period of time and, to the authors’ knowledge, no data is available on the variation of AHRS accuracy over time. However, the use of AHRS in biomechanics currently considers instrumented clinical evaluation for tests ranging from a few seconds (e.g. 3m Timed-Up & Go) to a few minutes (e.g. 10 minutes’ walk test) and their use is also foreseen as a solution for long-term monitoring of patients. The objective of this study is therefore to characterize the evolution of accuracy over time. Specifically, this study aims at documenting using a controlled bench test with an optical motion analysis system as gold standard: 1) the effect of time on absolute and relative accuracy of AHRS under various conditions of sustained motions; and 2) the different sources of errors affecting AHRS accuracy over time.

## Materials and Methods

### Attitude and Heading Reference Systems (AHRS) and Test Apparatus

**Fig 1:**
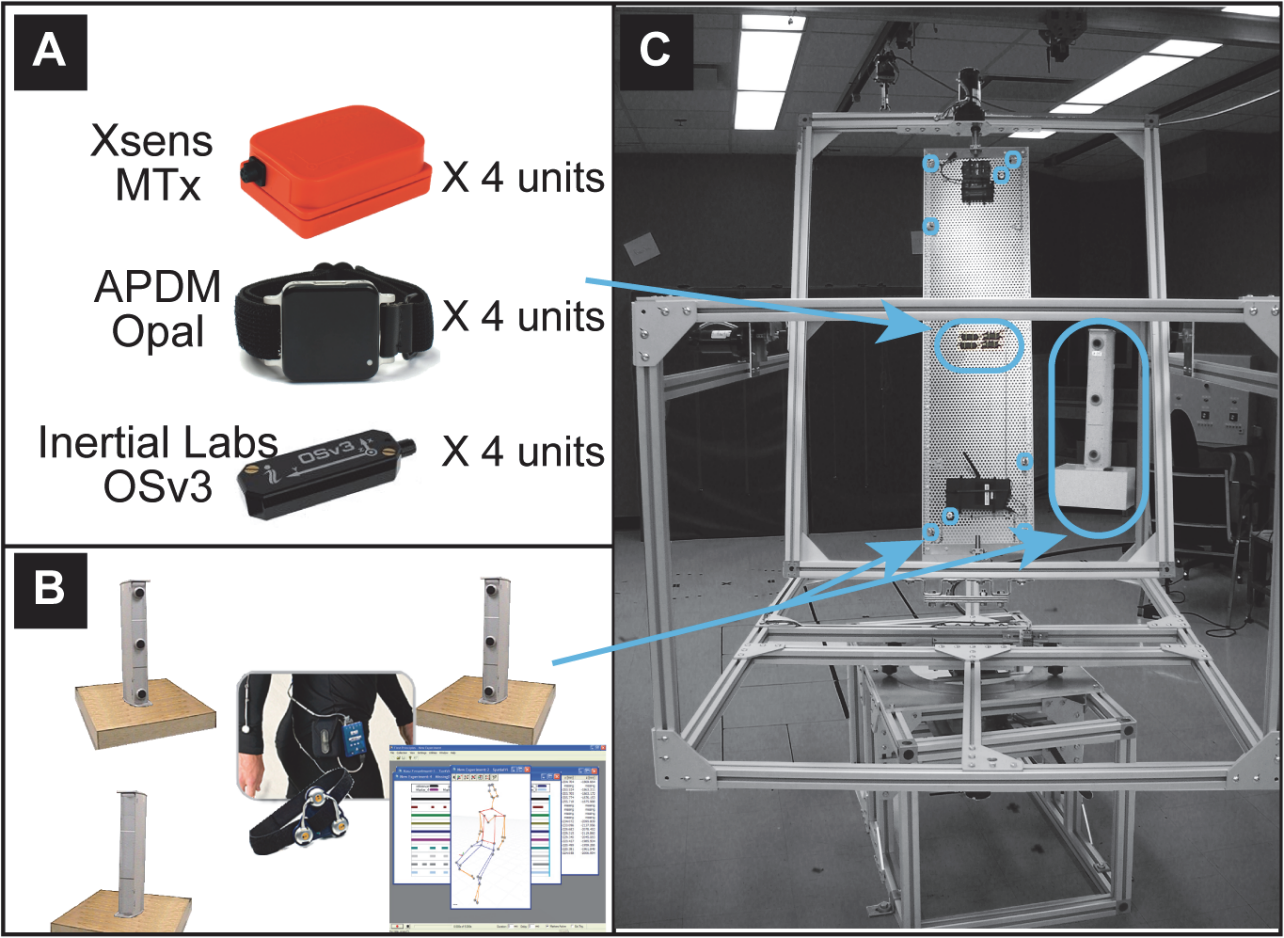
AHRS Accuracy Assessment Setup. (A) AHRS Selected for Evaluation (Xsens MTx, APDM Opal and Inertial Labs OSv3) (B) Optotrak Optical Gold Standard (C) Gimbal Table Setup with Payload.

Three commercially available AHRS (Xsens MTx [5], APDM Opal [6] and Inertial Labs OSv3 [7]) were selected for evaluation. Selected AHRS are shown in Fig. 1A. All three AHRS include 3-D accelerometers, gyroscopes and magnetometers within each module as well as a fusion algorithm, which uses the measurement obtained from all sensors to estimate the orientation of the module in a global reference frame based on gravity and magnetic North. Each manufacturer was contacted prior to the study to ensure all systems were set-up and used properly. In order to compare accuracy across AHRS, the experimental protocol consisted in applying controlled motions (speed and direction) within a standardized test scenario using an instrumented bench test (Fig. 1C). The bench test is comprised of a 3-axes Gimbal table, which allows single or multi- axes trajectories of motions for a payload attached to the center plate. The table is entirely made of aluminum and the impact of both the permanent magnets of the motors and the magnetic field induced with the motor powered-on were verified experimentally and were shown to be within magnetometers’ noise level 10cm prior to the location of the center of rotation (worst case). The center plate of the Gimbal table is also equipped with 16 active markers (8 on each side) from which a rigid body is created and tracked by an optical motion tracking system using 4 camera tours (Optotrak by Northern Digital), as illustrated in Fig. 1. The detailed description of the bench test appears in [4].

### Experimental Protocol

**Fig 2:**
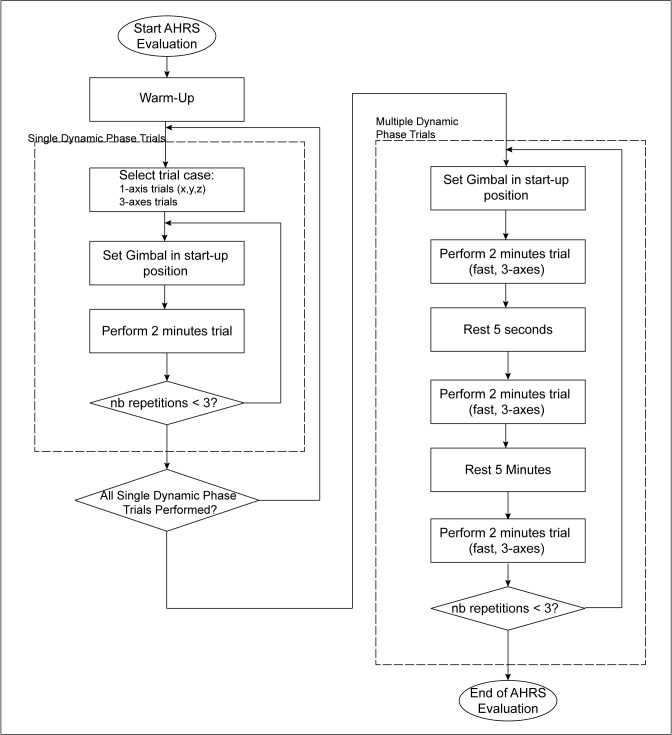
AHRS Accuracy Assessment Protocol.

The experimental protocol required four modules of the same type to be placed and visually aligned on the center plate of the Gimbal table as shown in Fig. 1C. The standardized test protocol presented in Fig. 2 was then used to evaluate each of the system separately. Briefly, the accuracy assessment protocol was initiated with a 5-minute warm-up period where modules were slowly rotated in three-directions. Then, a series of 2-minute dynamic conditions were performed at controlled velocities (≈ 90°/s), including 1-axis rotations (x, y and z individually) as well as 3-axes trajectories. Each condition, initiated from a standardized start-up position in an effort to minimize the possible effect of inertia on the trial’s repeatability, was repeated three times. Additional 3-axes trials were also performed at high speed (combined speed of ≈ 360°/s) in order to evaluate the ability of the different systems to stabilise and recover in time even when subjected to very demanding conditions. These *multiple dynamic phase trials* were composed of 3 dynamic periods separated by static pauses of various duration.

### Orientation Accuracy Evaluation

Accuracy evaluation is based on the underlying assumption that all AHRS modules as well as the central plate of the Gimbal table undergo the exact same motion throughout the trial. This assumption of equivalence is considered reasonable as the central plate was designed rigidly to minimize deformations. Specifically, each orientation measurement at time t is expressed in terms of a reference orientation, and movement is assessed from the global range of motion (ROM) using quaternions [4]. In order to cover accuracy evaluation for typical use of AHRS in biomechanics [8], the current study evaluates both absolute accuracy (accuracy of one AHRS module compared to a gold standard) and relative accuracy (accuracy between pairs of AHRS modules).

Absolute accuracy

Absolute accuracy refers to the ability of an AHRS module to measure variations in the orientation of a segment over time. Absolute accuracy is assessed by comparing the AHRS orientation measurement to those of an optoelectronic gold standard, here the Optotrak motion capture system from Northern Digital [9]. At a distance of 2.25m, the accuracy of the Optotrak 3020 is reported to be 0.1mm for the x and y coordinates and 0.15mm for z by the manufacturer. In the present study, the system was optimized to track the orientation of the center plate of the Gimbal table with a worst-case trueness estimated to 0.7° based on Monte Carlo analysis [4, 8]. Gold standard sampling rate was set to 100Hz and data were acquired with NDI First Principle v.1.2.4. To allow for comparison, AHRS data were resampled when required and synchronized to the reference data through cross-correlation. Data processing was performed using Matlab (v.7.12.0.635 (R2011a) from MathWorks). The mean difference in the ROM measured by the inertial system to the ROM measured by the gold standard ($\bar{ROM_{d}}$) is then computed using the first 60 seconds of the trial and then segmented into two 30 seconds time intervals (Ta = 0–30s; Tb = 30s–60s) in order to document the change in accuracy over time. Each condition was repeated three times and measured simultaneously by four modules of the same type, hence issuing 12 measurements per condition for both time intervals. After verification of the normality of the data using the Kolmogorov-Smirnov test, paired t-tests are used to evaluate the effect of time on orientation’s absolute accuracy.

Relative accuracy

Relative accuracy refers to the capability of a pair of modules to measure changes in relative orientation between them. In biomechanics, this can be concretely related to the capability to measure joint angles. Relative accuracy therefore involves both the ability of the modules to individually track the change in orientation experienced and their ability to express those orientation measurements in a matching reference frame. In the present study, multiple units of the same type, placed on the center plate of the Gimbal table, underwent the same motion at the same time. Based on the assumption of equivalence in the movement, the relative orientation of all pairs of modules should therefore remain constant throughout the trials. The mean variation in the relative orientation for a pair of module ($\bar{\Delta OR_{d}}$) is therefore computed for assessment of relative accuracy. Each trial being evaluated by four units of the same type simultaneously, six pairs of modules can be created. Considering each condition is repeated three times, a total of 18 measurements per condition are available for both time intervals (Ta = 0–30s, Tb = 30s–60s). Again, verification of the normality of the data through Kolmogorov-Smirnov allows for processing of the data using paired t-test to evaluate the impact of time on relative accuracy.

### System Robustness over Time

Bearing in mind that (1) the principal advantages of AHRS for biomechanics are their portability (not limited to a given capture volume) and their long-term recording possibilities, and that (2) magnetic perturbations are omnipresent in almost any environment, it is essential to study the capability of the systems to maintain a certain accuracy level over time. Such analysis goes beyond the study of the evolution of accuracy under sustained motion, looking into the ability of the systems to recover when static pauses are granted, and attempting to identify the source of the errors observed. This level of analysis is achieved using the multiple dynamic phases’ trials (i.e. fast 3-axes trials composed of three dynamic phases separated by static pauses of various lengths). The first step of the analysis consist in the assessment of the relative accuracy over the first 15 seconds of each dynamic phases, considering the beginning of the trial as the reference orientation in order to document the change in accuracy over time when static pauses are allowed. Then, relative accuracy is again computed over the first 15 seconds of each dynamic phases, but this time considering the phase initial static orientation as the reference orientation for each phase to emulate a reset of the dynamic estimation error. The final step makes use of the specific assumption that all modules were aligned on the Gimbal table. Variation in the measured initial orientation can therefore be attributed to a variation between the modules’ Inertial frame definition. Orientation data can then be corrected for the identified variation and the relative accuracy, assessed for the compensated data.

## Results

### Absolute Accuracy in Dynamic Conditions

For slow rotations, all systems have shown a mean absolute orientation accuracy below 5° for 60s trials, regardless of the direction of the rotation. Segmenting those 60s into two 30 second periods revealed a statistically significant (p<0.05) decrease in accuracy (i.e. increase in mean difference) across all systems for 3-axes trials. For single-axial trials, the impact of longer trials on absolute accuracy varied across the systems and the direction of motion (Xsens MTx has a significant time-effect for z rotations; APDM Opal, for x and y rotations; and Inertial Labs OSv3, for x-rotations). Details on absolute accuracy values are available in Table 1.

**Table 1:** Portrait of Absolute and Relative Accuracy Variation over Time.

		Xsens MTx		APDM Opal		Inertial Labs OSv3	
	Time Period	Absolute Accuracy	Relative Accuracy	Absolute Accuracy	Relative Accuracy	Absolute Accuracy	Relative Accuracy
*X rotation*	0–60s	0.6° (0.2°)	3.0° (1.0°)	4.6° (3.5°)	10.3° (5.4°)	1.0° (0.5°)	6.5° (2.4°)
	0–30s	0.5° (0.2°)	2.5° (0.7°)	2.6° (1.9°)	5.8° (3.0°)	0.8° (0.4°)	6.1° (2.2°)
	30–60s	0.6° (0.4°)	*3.5° (1.2°)	*6.4° (5.2°)	*14.7° (7.9°)	*1.3° (0.7°)	*7.0° (2.6°)
*Y rotation*	0–60s	0.5° (0.2°)	2.1° (0.6°)	3.6° (2.7°)	11.5° (7.1°)	2.5° (0.6°)	4.6° (1.6°)
	0–30s	0.4° (0.2°)	1.8° (0.4°)	3.1° (2.4°)	6.3° (4.1°)	2.6° (0.5°)	4.3° (1.4°)
	30–60s	0.6° (0.3°)	*2.4° (0.8°)	*4.2° (3.1°)	*16.7° (10.1°)	2.4° (0.8°)	*4.8° (1.7°)
*Z rotation*	0–60s	0.6° (0.2°)	2.6° (0.6°)	1.9° (0.8°)	4.1° (2.1°)	1.9° (0.6°)	5.4° (1.4°)
	0–30s	0.3° (0.1°)	1.9° (0.6°)	1.9° (0.8°)	2.5° (1.3°)	1.9° (0.6°)	5.3° (1.3°)
	30–60s	*0.8° (0.3°)	*3.2° (1.1°)	1.9° (1.1°)	*5.7° (3.1°)	1.8° (0.6°)	*5.6° (1.6°)
*3-axes rotation*	0–60s	2.0° (0.7°)	4.7° (1.8°)	3.8° (1.9°)	11.4° (5.9°)	2.5° (1.2°)	7.5° (3.6°)
	0–30s	1.0° (0.4°)	3.0° (0.7°)	1.2° (0.5°)	5.3° (2.5°)	2.0° (0.9°)	7.3° (3.1°)
	30–60s	*3.0° (1.1°)	*6.3° (3.0°)	*6.4° (3.4°)	*17.5° (9.7°)	*3.1° (1.6°)	7.8° (4.3°)

*Time effect on accuracy statistically significant with α = 0.05.

### Relative Accuracy in Dynamic Conditions

For slow rotations, mean relative orientation accuracy varied across the systems and conditions. While Xsens MTx remained under 5° for 60s trials regardless of the direction of the rotation, Inertial labs OSv3’s performance varied between 4° and 7.3° depending on the direction of the rotation, and APDM Opal scored slightly above 10° for all types of slow rotations over 60s except for z-rotation. Hence, results show an increase in mean difference values when evaluated using pairs of modules in comparison to those reported for absolute accuracy.

Analysis of the evolution of the accuracy through segmentation of the results into two 30 second intervals revealed a statistically significant effect of time (p<0.05) on accuracy for all systems and conditions except for Inertial Labs OSv3 3-axes trials. However, the extent of the effect varies across the systems and the conditions tested. While Inertial Labs OSv3 has shown a mean difference below 1.0° regardless of the type of motion performed, Xsens MTx presented a mean variation slightly higher, varying from 0.6° to 3.3°. APDM Opals relative accuracy was the most affected by time with a mean difference in relative accuracy varying between 3.2° and 10.4° for uni-axial conditions and increasing to 12.2° in multi-axes trials. Details on relative accuracy values are also available in Table 1.

### Systems Robustness over Time

**Fig 3:**
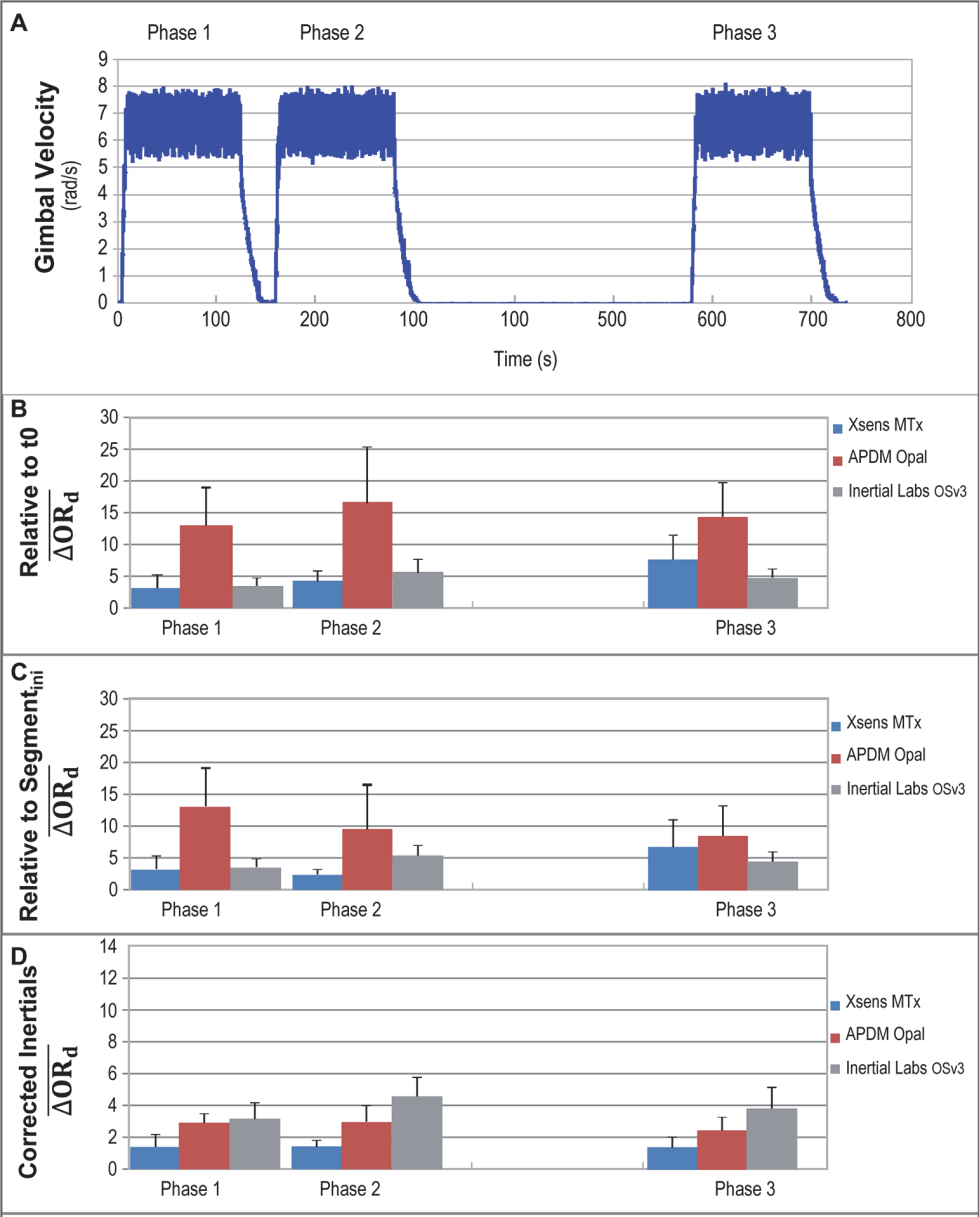
Change in Relative Accuracy over Time. Change in Relative Accuracy for Repeated Fast Dynamic Segments when Considering (B) the Initial Trial’s Orientation (t0) as the Reference Orientation, (C) the Initial Orientation of the Specific Dynamic Segment as the Reference Orientation and (D) the Initial Orientation Error as Due to a Difference in Inertial Definition.

System recovery was evaluated using relative accuracy measurement so to implicitly consider both dynamic estimation errors and inertial frame definition divergence between modules in the evaluation. The evolution of relative accuracy for all three systems over repeated 2 minutes dynamic segments is illustrated in Fig. 3. Part A of Fig. 3 illustrates the dynamic motion commanded to the Gimbal table. Part B of that same figure illustrates the mean relative error computed over all pairs of modules during the first 15 seconds of the three dynamic phase, using the relative static orientation at time t = 0 as the reference orientation. During phase 1, systems mean differences varied between 3° and 13° (Xsens MTx: 3.2°; APDM Opal: 13.1°; Inertial Labs OSv3: 3.6°). After a short 5 seconds stabilisation period, phase 2 was initiated. The mean differences observed during phase 2 increased for all systems (Xsens MTx: 4.3°; APDM Opal: 16.7°; Inertial Labs OSv3: 5.8°). The systems where then allowed to rest for 5 minutes prior to phase 3. Mean differences for phase 3 varied between 4° and 12° (Xsens MTx: 7.7°; APDM Opal: 14.4°; Inertial Labs OSv3: 4.7°).

For the results illustrated in part C of Fig. 3, change in relative orientation was assessed using the phase initial static orientation as a reference point to emulate a sporadic reset of the estimation error. Doing so, phase 2 and phase 3 mean errors decreased for all systems (Phase 2: 2.4° for Xsens MTx, 9.6° for APDM Opal, 5.3° for Inertial Labs OSv3; Phase 3: 6.8°, 8.6°, 4.4° for Xsens MTx, APDM Opal and Inertial Labs OSv3 respectively).

The last part of Fig. 3 again represents the mean difference on relative orientation computed over all pairs of modules using the phase initial static orientation as the reference point, but this time considering the initial difference in measured orientation as due to a difference in the inertial frame definition between the modules (since modules were aligned). Correcting for that initial difference allows to decrease the mean relative error below 5° for all systems, across all phases.

## Discussion

In slow motion, all systems evaluated for this study have shown acceptable single and multi-axes absolute angular motion accuracy under sustained conditions of motion when compared to reported accuracy and reliability of diverse traditional methods for biomechanical kinematics assessment [10, 11]. Indeed, all systems have shown a mean accuracy below 5° for 60s rotations. However, a closer look at the changes in the accuracy during that 60s period revealed a time effect more or less significant depending on the system and the direction of motion, with a worst-case drop in accuracy for all systems revealed for 3-axes sustained rotations (mean increase in difference of 2.0° for Xsens MTx, 5.2° for APDM Opal, 1.1° for Inertial Labs OSv3). Relative accuracy was, in general, even more affected over time than absolute accuracy, with a mean increase in difference varying between 0.6° and 3.4° for Xsens MTx, 3.2° to 12.3° for APDM, and 0.4° to 0.9° for Inertial Labs OSv3, depending upon the type and the direction of the motion. Orientation estimation accuracy with AHRS depends upon the ability of the system to recognize true motion from sensor-noise and environmental changes. While sensor-noise, defined as a combination of white noise and drift, has a direct effect on the quantity of the motion estimated, environmental changes affect the definition of the Inertial frame itself, which in turns helps in correcting the sensor drift. Indeed, a change in magnetometer measurement may be related to a change in direction, but it may also mean that the magnetic environment changed, which can translate into a need to adapt the Inertial frame to better fit this new environment. When modules are used in pairs, the correspondence in the independently-defined Inertial frames becomes even more important. The increase in mean differences on relative accuracy compared to absolute accuracy (thus decreased accuracy) are in line with Picerno et al. [1] observation’s that even closely placed and aligned AHRS may define their orientation differently. Indeed relative accuracy of AHRS is related to the ability of all modules to *locate* and *track* the exact same reference frame, regardless of the magnetic environmental perturbations experienced individually. The observation about time effect being more significant on relative accuracy than on absolute accuracy also emphasizes the importance of ensuring correspondence in Inertial frames definition, especially in a clinical biomechanics context where AHRS are used to look at changes in joint angles over time. It also suggests that for some systems, such divergence in the definition of the Inertial frame may even be considered as the main contributors in accuracy decrease over time.

To test for this hypothesis, we conducted the 12-minute tests comprised of three high-speed dynamic phases separated by static pauses. This test was designed to evaluate the capability of the systems to maintain and/or recover good accuracy over time, as well as to identify the different sources of the errors observed. During those tests, all three systems have shown increases in mean relative differences between the first and the second dynamic phases (Phase 1: 3.2°, 13.1° and 3.6° for Xsens MTx, APDM Opal and Inertial Labs OSv3; Phase 2: 4.3°, 16.7° and 5.82° for Xsens MTx, APDM Opal and Inertial Labs OSv3). However, this difference can be corrected for all systems when difference in orientation is computed considering the phase initial orientation as a reference. Such results suggest that the difference in performance seen between phase 1 and phase 2 is mainly due to a dynamic estimation error that built up over time. In addition, the systems were incapable of recovering by themselves from the error accumulated in phase 1 with the short 5-second pause. The error shown during the third phase is however different between the systems. While two out of the three systems perform better during phase 3 when compared to phase 2 (APDM Opal and Inertial Labs OSv3), the Xsens MTx shows an increase in the mean relative error between those two phases. Such results suggest something else apart dynamic estimation error is affecting the data.

In this specific study, since modules were aligned on the center plate of the Gimbal table, it is possible to verify the effect of the initial Inertial coordinate system definition on relative accuracy by mathematically compensating for it. Doing so, all three systems improved their mean relative accuracy throughout the different phases below 5°. This test confirms that the changes of accuracy of orientation measurement from AHRS over time depends upon two main sources of errors which are closely related to each other and for which the robustness varies across the systems. These sources are the magnetic environment around the modules (or the ability of the modules to locate and track the same Inertial frames regardless of those variations) and the dynamic estimation error.

Based on clinical data, such as lumbar spinal mobility reported by Saidu et al. [12], the mean accuracies obtained for all systems for slow motion rotation reveals the possibility to use the studied AHRS for evaluation of coarse biomechanical features of a single segment, such as trunk inclination variation, over sustained conditions of motion up to 60 seconds. However, the lower mean relative accuracy observed for all three systems, compared to their mean absolute accuracy, and their significant decrease over time for two of the three systems (Xsens MTx and APDM Opal) suggest caution when using AHRS in pairs to compute joint angle motions during sustained condition of motion (ex: knee range of motion during sustained walking). As identified with the 12-minute test (12MDP), relative accuracy is affected by a difference in the definition of the Inertial reference frame. Initiating the experiment in a stable “clean” magnetic environment would certainly improve these results. In this study, although a 0.5m clearance with any ferrous material was respected in the setup, the Gimbal table was placed in a standard biomechanical lab, which is believed to have some small variations in the magnetic environment. Considering magnetically-clean environment is not always easy to find, clinically-reliable AHRS orientation measurements therefore rely upon some type of initial AHRS’ environment characterization or correction. Based on the results obtained with the 12MDP tests, the authors believe that having an external measurement of the initial relative orientation of the modules would allow for Inertial reference systems correction and therefore increase relative accuracy and robustness of all systems over a 60s period and longer. Indeed, the ***corrected*** relative accuracy reported for all evaluated AHRS under the demanding Gimbal conditions is close to the accuracy reported for various methods for joint angle measurement [10, 11, 13] used as outcomes in clinical applications and decisions [14, 15].

## Conclusion

The main objectives of this study were to provide an independent evaluation of market-available AHRS performance and to determine the robustness of the systems to be used for longer recordings corresponding to less constrained clinical biomechanics scenarios. The mean 5° absolute accuracy obtained for 60s slow sustained condition of motion and the ***corrected*** mean 5° relative accuracy reported for sustained motion allows the authors to conclude that AHRS is truly an attractive solution for mobility evaluation in clinical setup when compared to reported accuracy and reliability of diverse traditional methods for biomechanical kinematics assessment [10, 11]. Furthermore, the low cost and portable features of AHRS increases the accessibility to clinical quantitative assessment of mobility features and make AHRS good candidates for real-life mobility evaluation, although the robustness of orientation accuracy with longer period of time also will need to be appraised.

This paper specifically focussed on the assessment of the change in accuracy over time. While it was shown that part of the estimation errors induced with time may be corrected using a closer in-time reference point to reset the data, this paper also shown that part of the error is due to the ability of the modules to locate and track the exact same Inertial reference frame. Initial alignment of the different Inertial frame led to a 5° mean relative accuracy for all systems at high speed over three distinct phases of dynamic motion, suggesting the necessity to make a connection between the modules environment definition.

Moreover, the specific performances of different market-available systems reported in this paper are based on controlled conditions performed on the Gimbal table. Such controlled conditions were required to identify the limits of the AHRS technology itself as well as to be able to compare the robustness of the different systems. However, the continuous motion imposed by the scenarios used with the Gimbal table compared to human motion, which can be assumed to have a zero-mean acceleration and angular speed over a certain period of time, imposed the most difficult conditions of use for AHRS. As a result, accuracy of AHRS may be better than those reported in this study under human condition of motion. However, it is clear from the present results that continuous recording using AHRS will require further improvements in fusion algorithms so that magnetic compensation is optimized for longer recording periods.
